# Supplementary material for: Candidacy for Cochlear Implantation in Prelingual Profoundly Deaf Adult Patients
Source: J Clin Med. 2022 Mar 28;11(7):1874. doi: 10.3390/jcm11071874 (PMC8999851; doi:10.3390/jcm11071874)
Supplement: Supplementary file 1 [file jcm-11-01874-s001.zip › jcm-1612132-supplementary.pdf]

Table S1 : Socio-professional levels according to Poitrenaud’s scale

Level	Description
1	No diploma and illiterate
2	No diploma, knows how to read, write and count
3	Professional capacity diploma primary school qualification
4	Professional capacity diploma after secondary school qualification
5	Bachelor's degree
6	Post-secondary short (2 years maximum after bachelor degree)
7	Post-secondary long (3 years or more after bachelor degree)

Table S2: The Category of Auditory Performance (CAP) scale

Score	Description
1	No awareness of environmental sound
2	Awareness of environmental sounds
3	Recognizes environmental sounds
4	Responds to speech sounds
5	Discriminates at least two speech sounds
6	Understands common phrases without lipreading
7	Understands conversation without lipreading with a familiar talker
8	Can use the telephone with a familiar talker
9	Can use the telephone with an unfamiliar talker

Table S3: The Speech Intelligibility Rating scale (SIR) scale

Category	Description
1	Pre-recognisable words in spoken language.
2	Connected speech is unintelligible but is developing for single words.
3	Connected speech is intelligible to a listener who concentrates and lip reads within a known context.
4	Connected speech is intelligible to a listener who has little experience of a deaf person’s speech. The listener does not need to concentrate unduly.
5	Connected speech is intelligible to all listeners. The child is easily understood in everyday contexts
